# Versatility of the complement system in neuroinflammation, neurodegeneration and brain homeostasis

**DOI:** 10.3389/fncel.2014.00380

**Published:** 2014-11-07

**Authors:** Franca Orsini, Daiana De Blasio, Rosalia Zangari, Elisa R. Zanier, Maria-Grazia De Simoni

**Affiliations:** ^1^Department of Neuroscience, IRCCS – Istituto di Ricerche Farmacologiche Mario NegriMilan, Italy; ^2^Department of Experimental and Clinical Sciences, University of ChietiPescara, Italy; ^3^Department of Anesthesia and Critical Care Medicine, Fondazione IRCCS Ca’ Granda Ospedale Maggiore Policlinico, University of MilanMilan, Italy

**Keywords:** complement system, therapeutic targets, endothelium, stroke, traumatic brain injury, Alzheimer’s disease, brain homeostasis

## Abstract

The immune response after brain injury is highly complex and involves both local and systemic events at the cellular and molecular level. It is associated to a dramatic over-activation of enzyme systems, the expression of proinflammatory genes and the activation/recruitment of immune cells. The complement system represents a powerful component of the innate immunity and is highly involved in the inflammatory response. Complement components are synthesized predominantly by the liver and circulate in the bloodstream primed for activation. Moreover, brain cells can produce complement proteins and receptors. After acute brain injury, the rapid and uncontrolled activation of the complement leads to massive release of inflammatory anaphylatoxins, recruitment of cells to the injury site, phagocytosis and induction of blood brain barrier (BBB) damage. Brain endothelial cells are particularly susceptible to complement-mediated effects, since they are exposed to both circulating and locally synthesized complement proteins. Conversely, during neurodegenerative disorders, complement factors play distinct roles depending on the stage and degree of neuropathology. In addition to the deleterious role of the complement, increasing evidence suggest that it may also play a role in normal nervous system development (wiring the brain) and adulthood (either maintaining brain homeostasis or supporting regeneration after brain injury). This article represents a compendium of the current knowledge on the complement role in the brain, prompting a novel view that complement activation can result in either protective or detrimental effects in brain conditions that depend exquisitely on the nature, the timing and the degree of the stimuli that induce its activation. A deeper understanding of the acute, subacute and chronic consequences of complement activation is needed and may lead to new therapeutic strategies, including the ability of targeting selective step in the complement cascade.

## The complement system: a background

In 1891, Buchner et al. discovered and reported a heat labile factor able to kill bacteria in blood, naming it “alexin” (in Greek, means “to ward off”) (Ehrlich and Morgenroth, 1899; Buchner, 1981; Nesargikar et al., 2012). Bordet subsequently demonstrated that immune lysis requires the presence of two factors: a heat-labile lytic factor similar to “alexin” and a heat-stable factor, which he termed “sensitizer” (now known to be the antibody) (Morgan, 1990; Nesargikar et al., 2012). In 1899, Paul Ehrlich refined this theory describing the requirement for a supplementary molecule, named “the complement” (which replaced the historical term “alexin”) necessary to induce antibody-induced bacterial lysis. Over one hundred years later, the role of the complement system is known to extend well-beyond that of a supplementary molecule. This physiological system is now recognized as an extremely potent component of the defense cascade of innate immunity, able not only to scavenge pathogens and antigens but also to respond to endogenous so called danger signals. Numerous experimental and clinical studies have recently demonstrated the involvement of this system in different acute and chronic pathological conditions, highlighting the importance of complement as a crucial factor in the activation and control of the generalized immune response. In addition, beyond elimination of potentially toxic molecules, its contribution to diverse homeostatic processes, such as lipid metabolism, angiogenesis, tissue modeling and maintenance has been recently proposed.

**Figure 1:**
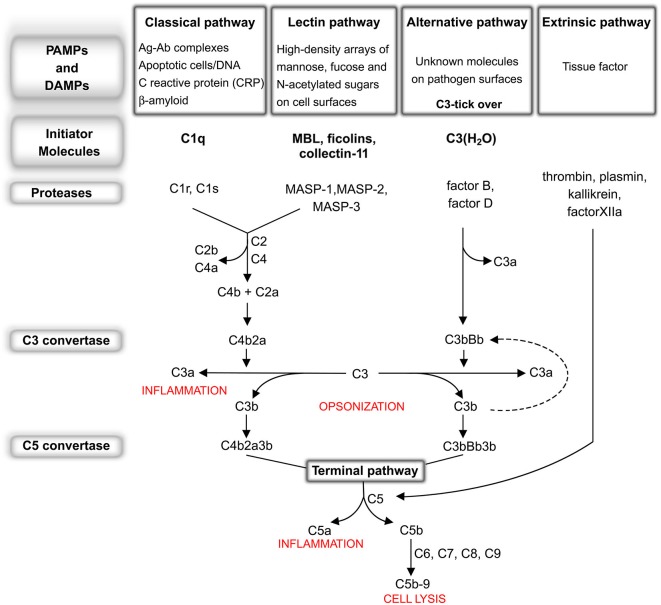
**The complement system: an overview. Classical pathway (CP):** C1q, the CP initiator, recognizes and binds antigen-antibody complexes or specific molecules, including β-amyloid, C reactive protein (CRP), DNA and apoptotic bodies. After binding, the C1r and C1s proteases subsequently cleave C4 and C2 to generate C4a, C4b, C2a, C2b, permitting the formation of C4b2a (CP C3 convertase). This complex cleaves C3 into C3a, which, in turn, acts as potent anaphylatoxin, and C3b that binds to the complex forming the C4b2a3b protein block (CP C5 convertase). **Lectin pathway (LP):** MBL, ficolin-1, ficolin-2, ficolin-3 and collectin-11, the LP initiators, recognize and bind high-density arrays of mannose, fucose and N-acetylated sugars exposed by pathogens or by self-altered cells. After binding, the MBL-associated serine proteases (MASPs), MASP-1 and -2, associated in complex with the above recognition molecules (MBL/ficolins), cleave C4 and C2, thereby forming C3 convertase and C5 convertase in a similar manner to that of the CP (Ehrnthaller et al., 2011). The role of the third serine protease, called MASP-3, remains unclear (Kjaer et al., 2013). **Alternative pathway (AP):** the activation of the AP is driven by a spontaneous hydrolysis of circulating C3 (called tick-over process) to form C3(H_2_O). This molecule then associates factor B and factor D to form C3bBb (AP C3 convertase). Similar to the C3 convertase generated in the CP and LP, this complex splits C3 into C3a and C3b, with the latter creating a new C3 convertase (AP amplification loop, dotted line), and/or binding C3 convertase already present to create the C3bBb3b complex (AP C5 convertase). **Extrinsic pathway:** the recent characterization of this activation pathway suggests that it is driven by activated proteolytic enzymes, including thrombin, plasmin, kallikrein, factor XIIa. Thrombin possesses its own C5 convertase activity and, under undefined conditions, has been shown to have the capacity to directly cleave C5 to generate the correspondent active fragments (Huber-Lang et al., 2006). Recently, it has been shown that the coagulation serine proteases are likewise able to cleave C3 (Markiewski et al., 2007; Amara et al., 2010). **Terminal pathway:** CP, AP, LP and the extrinsic pathway all converge at C5 convertase formation, activating a common cascade through the cleavage of C5 into the anaphylatoxin C5a and the active C5b. Finally, (1) C3b fragment binds the targeted cell allowing the assembly of C6, C7, C8 and C9 in a pore called membrane attack complex (C5b-9 or MAC), that causes the direct lysis of the cell; (2) many fragments, such as C3b and C4b as well as C5b, work as opsonins triggering an overactivation of the phagocytic response; (3) altogether complement components are able to orchestrate an adaptative immune reaction by communicating with multiple immune cells (Ricklin and Lambris, 2007) through different receptors, leading to a robust local and systemic inflammatory response.

The complement system consists of more than 30 fluid-phase and cell-associated proteins (Wagner and Frank, 2010), each with different functions including, but not limited to, initiator molecules, substrates, regulators, inhibitors and receptors for complement proteins. The activation of the complement system can be triggered by exogenous and/or endogenous danger signals (pathogen-associated molecular patterns—PAMPs and/or damage-associated molecular patterns—DAMPs respectively) through the classical (CP), lectin (LP) or alternative pathway (AP). These pathways are each activated by different types of danger signals but share the same cascade-like activation system consisting of a number of proteolytic reactions, during which an inactivated protein is cleaved into smaller and active peptide fragments (see Figure 1). Briefly, the CP is activated through the binding of C1q to antigen-antibody complexes or directly by specific molecules including β-amyloid, C reactive protein (CRP), DNA and/or apoptotic bodies. The LP is activated through different pattern recognition receptors, including, in humans, mannose binding lectin (MBL), ficolin-1, ficolin-2 and ficolin-3 and collectin-11 (CL-11). These lectin molecules bind to high-density arrays of mannose, fucose and N-acetylated sugars exposed by pathogens or by altered host cells. The activation of the AP is driven by the spontaneous hydrolysis of circulating C3 (tick-over process) into C3(H_2_O) on cellular surfaces. In addition, another complement activation pathway, named the extrinsic pathway, that is driven by serine protease components of the coagulation system, has been described (Huber-Lang et al., 2006). Increasing evidence suggests an intimate but not yet fully disclosed interaction between the complement and coagulation cascades which is particularly relevant in cerebrovascular disease, in which the balance between coagulation and fibrinolysis may be clinically manipulated for therapeutic purposes. All these pathways, converging on the C5 convertase formation (see Figure 1), activate a common cascade (named the terminal pathway, TP) through the cleavage of C5 into C5a and C5b. The former, along with C3a (generated by up-stream cleavage of C3), functions as anaphylatoxins, inducing a potent inflammatory response and stimulating the recruitment of peripheral immune cells. The latter fragment (C5b) binds to the targeted cell, allowing the assembly of C6, C7, C8 and C9 into a pore called membrane attack complex (C5b-9 or MAC), that causes the direct cellular lysis. Another critical consequence of complement activation is that many of these cleavage products, such as C3b and C4b as well as C5b, work as opsonins to trigger an overactivation of the phagocytic response. In addition, complement components are able to orchestrate an immune reaction by communicating with multiple immune cells through different receptors, thereby leading to robust local and systemic inflammatory responses (Ricklin and Lambris, 2007).

The complement system is also endowed with highly sophisticated regulatory mechanisms that serve to finely tune its physiological function (Ricklin and Lambris, 2007). The complement regulatory molecules are classified as fluid-phase or membrane-bound regulators (Table 1). One of the most well-studied circulating complement regulators is C1 inhibitor (C1-INH), a serine-protease that acts to inactivate the C1q/C1r/C1s and the MBL/MASP-1/MASP-2 complex. At high concentrations, C1-INH may also inactivate C3b generated by the AP (Wagner and Frank, 2010) as well as molecules associated with the kinin, fibrinolytic and coagulation systems (e.g., factor XII and IX) (Ehrnthaller et al., 2011).

**Table 1:** **Main complement regulators**.

Regulator	Abbreviation	Target pathway	Functions
**Fluid phase regulators:**
C1-inhibitor	C1-INH	CP and LP	Inhibits C1r, C1s, MASP-1 and MASP-2 proteolytic activities
MBL/ficolin-associated protein 1	MAP-1	LP	Binds MBL and ficolins and inhibits C4 deposition
Factor I	FI	CP, LP and AP	Cleaves C3b and C4b in their inactive fragments
C4 binding protein	C4BP	CP and LP	Accelerates decay of classical and lectin C3 convertase along with FI
Factor H	FH	AP	Accelerates decay of alternative C3 convertase along with FI
Carboxypeptidase N		CP, LP and AP	Inactivates C3a and C5a anaphylatoxin
**Membrane-bound regulators:**
CR1		CP, LP and AP	Binds C3b,C4b and C1q to promote phagocytosis of immune-complexes and accelerates decay of convertases
CD46		CP, LP and AP	Binds C3b and C4b accelerating decay of C3 convertase, cofactor for FI
CD55		CP, LP and AP	Accelerates decay of C3 convertases
Complement receptor 1-related protein y	Crry	CP, LP and AP	Cleaves C3b and C4b inhibiting C3 convertases formation, cofactor for FI
CD59		CP, LP and AP	Binds C8 and C9 preventing the assembly of C5b-9 lytic complex

*CP, classical pathway; LP, lectin pathway; AP, alternative pathway*.

The complement system also comprises a wide array of specific receptors for complement proteins by which the system induces phagocytosis and triggers the inflammatory response via direct communication with immune cells (Ricklin et al., 2010). The most important complement receptors include: (1) C1q receptors (gC1qR, C1qRp and cC1qR) that have been associated with the phagocytic process; (2) the integrin receptors, including complement receptor 3 (CR3, also known as CD11b/CD18) and 4 (CR4, also known as CD11c-CD18) that bind to iC3b fragment (a complement product coming from a further cleavage of C3b) to further promote cellular phagocytosis, cytokine responses, leukocyte trafficking and synapse formation; (3) C3a (C3aR) and C5a (C5aR or CD88) receptors that trigger a sustained proinflammatory signaling. Overall the role of the complement receptors is still to be fully elucidated.

## Sources and compartments of activation

### Sources in health and brain injury

Complement proteins account for approximately 4% of total blood proteins. Physiologically, they monitor the blood and the cell surfaces and are constantly primed for activation when potential threats are detected. The liver represents the major source of complement components released into the systemic circulation, and hepatocytes are known to be capable of synthesizing the myriad classes of complement proteins (Morgan and Gasque, 1997; Brennan et al., 2012). Other cell types, such as fibroblasts, monocytes, epithelial and endothelial cells and, most notably, all brain cell populations, are programmed to produce selected complement factors. The adult brain, once considered immunologically privileged, is subjected to considerable immune surveillance and possesses its own immune competence. Current evidence suggests that astrocytes, neurons, microglia and oligodendrocytes are all able to directly synthesize several factors, regulators and receptors for complement proteins (Woodruff et al., 2010; Veerhuis et al., 2011). Neurons, astrocytes and microglia can produce complement initiators such as C1q (Stevens et al., 2007) and C3, receptors such as C3aR and C5aR as well as inhibitors such as C1-INH and CD59, although other complement proteins can be specifically produced by the different brain cells. Under normal conditions, complement synthesis in brain cells is low and is believed to be involved in physiological processes during brain development and homeostasis (vide infra). Following cellular injury and damage, complement synthesis in brain cells can markedly increase and contribute to tissue damage (Woodruff et al., 2010). The activation of the complement system has been extensively demonstrated in both chronic neurodegenerative and acute neuroinflammatory central nervous system (CNS) conditions, with neurons showing selective vulnerability to complement mediated damage (Singhrao et al., 2000), most likely due to their low basal expression of cell-membrane associated complement regulators (e.g., CD55 and CD46). However, distinctive kinetics of complement activation between chronic neurodegeneration and acute brain injury and neuroinflammatory insults occur. In neurodegenerative disorders, local complement biosynthesis and uncontrolled complement activation in the tissue are crucial for contributing to neuronal loss and local inflammation, with blood brain barrier (BBB) injury appearing during the disease chronic stages (Gasque et al., 2000). Conversely, one common feature of acute brain injuries, such as stroke, subarachnoid hemorrhage and brain trauma, includes early and severe BBB breakdown. Thus, in addition to complement proteins and regulators produced locally in the tissue, brain parenchyma immediately following acute injury is rapidly invaded by a number of inflammatory cells and molecules, including complement proteins circulating in high concentration in blood (Brennan et al., 2012). Although this process is essential for triggering the removal of cellular debris, when over-activated, it may severely affect neuronal and glial integrity in cells spared at the time of the primary injury.

### The brain endothelium: a critical compartment of action

**Figure 2:**
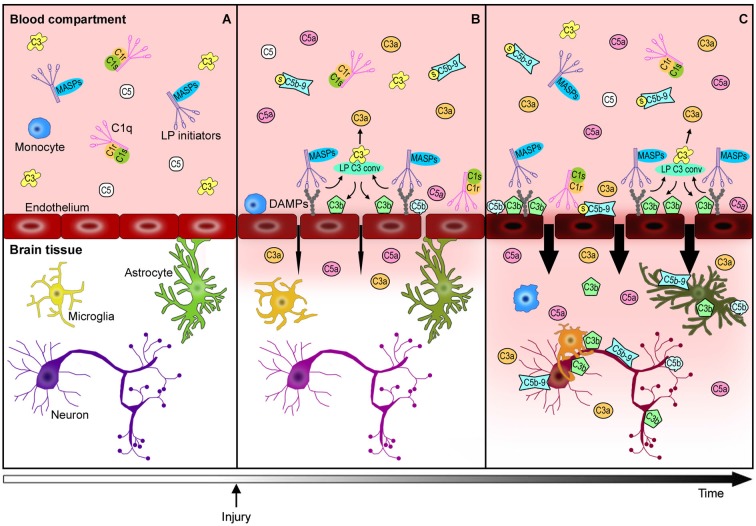
**Complement-mediated endothelial damage: working hypothesis**. Healthy brain endothelial cells physically isolate the brain parenchyma from the blood compartment. Under physiological conditions, complement proteins, such as C1q, LP initiators, C3 and C5, monitor the blood and the cell surfaces for potential threats **(A)**. The additional complement factors that normally circulate in the bloodstream, have been omitted in this figure for purposes of clarity and simplification. When a cerebrovascular injury occurs **(B)**, endothelial cells may change their glycosylation profile, leading to the exposure of high density arrays of sugar (DAMPs). LP initiators, through their carbohydrate recognition domains, can recognize and bind altered endothelial cells, leading to LP complement activation **(B)**. Complement activated fragments trigger the expression of adhesion molecules and the release of chemokines and cytokines (not shown) on endothelial cells, resulting in the worsening of the BBB leakage (**B→C**). The injured brain parenchyma is then rapidly invaded by the full immune arsenal, including the complement proteins, cytokines and immune cells (e.g., monocytes) belonging to the blood compartment, leading to amplification of local damage **(C)**. The overactivation of the complement system in brain tissue leads to: (1) a potent inflammatory response and the recruitment of peripheral immune cells mediated by C3a and C5a anaphylatoxins, (2) direct lysis of neurons and other brain cells, including those that are potentially savable, by C5b-9, (3) opsonization with subsequent microglia/macrophage phagocytosis of the target cells by C3b and C5b. This schema proposes a key role for endothelial cells in triggering local LP complement activation and suggests that targeting the peripheral compartment may represent effective strategy for brain protection from injury and different acute CNS diseases.

Brain vascular endothelial cells are major players in this cascade of events. They represent the physical barrier between the periphery and the brain parenchyma and since they are continuously exposed to blood-derived complement effectors, they remain a target of circulating active complement molecules (Bossi et al., 2011). In response to stress signals, endothelial cells may expose DAMPs to become selective targets for complement initiators. Endothelial cells can also produce selected complement components, subsequently increasing the local availability and cell toxicity of these molecules. The complement-mediated endothelial damage promotes BBB leakage with a subsequent influx of active complement fragments and cytokines, as well as immune cells into the injured brain tissue with amplification of local inflammation (Figure 2). Endothelial cells may therefore play a central role in triggering/modulating local complement activation and in the partition of complement components from blood to brain.

C1q is known to directly bind endothelial cells (Yin et al., 2007; Bossi et al., 2008) inducing the expression of adhesion molecules, such as E-selectin, intercellular adhesion molecule-1 (ICAM-1) and vascular cell adhesion protein-1 (VCAM-1; Lozada et al., 1995), and the release of chemokines and cytokines, such as IL-8, monocyte chemotactic protein-1 (MCP-1) and IL-6 (van den Berg et al., 1998). It has been reported that C1q and MBL compete for the binding to endothelial surfaces (Oroszlán et al., 2007). MBL deposition on endothelial cells has been demonstrated independently from C1q, both in *in vitro* and *in vivo* settings and is known to occur after oxidative stress on human umbilical vein endothelial cells (HUVECs), accompanied with iC3b deposition. Furthermore, MBL and iC3b deposition are reduced in the presence of functionally inhibitor anti-MBL antibodies (Collard et al., 2000) suggesting that MBL adhesion on endothelial cells mediates complement activation. It has also been proposed that MBL deposition is driven by increased endothelial expression of cytokeratin 1 after hypoxia, since anti-cytokeratin treatment is able to attenuate MBL and iC3b presence on HUVECs under the same conditions (Collard et al., 2001). Although the specific MBL-binding molecules expressed by damaged endothelial cells remain unclear, MBL deposition has been demonstrated *in vivo* in different organs, such as heart (Pavlov et al., 2012), kidney (Møller-Kristensen et al., 2005; Castellano et al., 2010), intestine (McMullen et al., 2006) and brain (Gesuete et al., 2009; Orsini et al., 2012) following ischemia reperfusion injury. MBL deposition also occurs after both human and experimental traumatic brain injury (TBI; Longhi et al., 2014), suggesting that this response represents one of several toxic events after trauma (Figure 2). Another protein belonging to the LP, the serine protease MASP-1, is currently under evaluation for its effect on endothelium. It has been demonstrated that MASP-1 induces p38- mitogen-activated protein kinases (MAPK) activation, NFkappaB signaling and Ca^2+^ mobilization in HUVECs, inducing IL-6 and IL-8 production (Jani et al., 2014) as well as E-selectin expression (Dobó et al., 2014). MASP-1, like thrombin, also activates endothelial cells directly by cleaving the protease activated receptors (PARs; Megyeri et al., 2009). Other complement components have been demonstrated to interact with endothelial cells and promote vascular toxicity. For example, anaphylotoxin C3a and C5a may interact with the corresponding receptors on endothelial cells (Van Beek et al., 2000) inducing cytoskeletal modifications (Schraufstatter et al., 2002; Bossi et al., 2011) and increasing mRNA levels of IL-8, IL-1β and RANTES (regulated on activation normal T cell expressed and secreted) (Monsinjon et al., 2003). In addition, C3 knock-out (C3−/−) mice showed reduced immune complex-mediated vascular leakage when compared to wild type (WT) mice (Lister et al., 2007; Bossi et al., 2011), even though a direct toxic effect of C3a on endothelial cells has not been demonstrated (Schraufstatter et al., 2002). The same authors reported that C5a induces endothelial cells shrinkage with subsequent increased vascular permeability. The vascular properties of C5a have also been reported in an endotoxin-induced permeability model, in which C5aR-siRNA was able to limit the vascular leakage (Liu et al., 2010). In addition to the effects on the aforementioned complement components, TP proteins can lead to vascular dysfunction. For example, the circulating soluble C5b-9 (sC5b-9), a cytotoxic inactive complex formed by C5b-9 terminal lytic complex associated with soluble regulators, induced vascular leakage on HUVECs and in an *in vivo* model of mesenteric microvessel permeability (Tedesco et al., 1997; Bossi et al., 2004).

Taken together, these data suggest that protein products derived from complement activation can act on endothelial cells and shift, via different mechanisms, endothelial activation towards a toxic phenotype with associated increased vascular permeability.

## The disruptive overwhelming power of complement in acute neuroinflammatory brain conditions

Stroke and TBI represent two major types of acute neuroinflammatory brain conditions. Stroke remains the third leading cause of death worldwide and the first cause of long-term disability in Europe (Corbyn, 2014), while TBI remains the principal cause of death and disability in active young adults today (Lingsma et al., 2010). Moreover, TBI has been proposed to be an independent risk factor for stroke (Burke et al., 2013). Fewer than 10% of stroke patients are eligible for treatment with tissue-type plasminogen activator, the only current therapy available, to date. Despite the creation of increasingly widespread networks of organized acute stroke units, a high proportion of hospitalized stroke patients survive with permanent neurological impairment and disability. Similarly, no treatment is currently available for TBI and surviving patients often report severe and/or prolonged disabilities. Although stroke and TBI share important molecular and cellular pathogenic mechanisms leading to the progression of damage, due to the different nature of the primary insult, they are also associated with specific cellular vulnerability and activation of distinct pathogenic cascades. We will focus on these two conditions that induce profound alterations of both central and peripheral immune system (Bellavance and Rivest, 2012; Chamorro et al., 2012), review the available data on the disruptive overwhelming power of complement in acute brain injury and summarize both the common and divergent relevant cellular and molecular mediators which represent candidates for the development of novel targeted therapeutic strategies.

### Stroke

#### Evidence of complement system activation

A role for the complement system in ischemia/reperfusion injury was first suggested by Hill and Ward (1971), who demonstrated the presence of C3-cleavage factors with chemotactic activity in damaged rat myocardial tissue. Activation of the complement system after ischemia/reperfusion injury in different organs such as heart, kidney, intestine and brain has been subsequently documented (Arumugam et al., 2009; Cervera et al., 2010; Orsini et al., 2012), suggesting that complement represents a common but key mechanism involved in the exacerbation of tissue damage after ischemia.

In stroke patients, activation of the complement system has been documented in plasma or serum samples as well as in post-mortem brain tissues. Specifically, C3a and C5a plasma levels were shown to increase post-stroke, reaching peak values at days 1 and 14 respectively (Mocco et al., 2006b). Follow-up studies have extended these observations, documenting an increase in C3 and C3a plasma levels in patients with small vessel disease or cardioembolic stroke when compared to controls, and additionally demonstrating a positive correlation between their levels and unfavorable outcome in cardioembolic stroke (Stokowska et al., 2013). Acute (day 1–2) plasma sC5b-9 levels in patients with either ischemic or hemorrhagic stroke have been shown to be significantly increased when compared to non-stroke controls (Széplaki et al., 2009; Zanier et al., 2014) and shown to increase over time during post-stroke recovery (Pedersen et al., 2004). In a cohort of mild stroke patients, Mocco et al. (2006b), observed decreased plasma sC5b-9 levels when compared to non-stroke controls. These data highlight that interpretation of alterations in systemic concentrations of complement factors is not straightforward, since injury severity or timing issues may markedly affect plasma dynamics of complement activation products. Furthermore, caution must be observed when interpreting the clinical data, since both an increase and decrease of circulating complement factors may be regarded as indicator of complement activation reflecting either increased synthesis or increased consumption/deposition.

In addition to the analysis of circulating factors, the presence of differing complement proteins in post-mortem brain tissues of human stroke patients has also been investigated, providing important information regarding the specific cell population associated with the expression or deposition of selective factors. Immunohistochemical analysis of human brain tissue has revealed that expression of factors, such as C1q, C4d, C3c and C9, is detectable in neurons in ischemic brain regions but absent in non-stroke control tissues (Pedersen et al., 2009). C1q immunostaining was associated with microglial cells, while C3c and C9 staining were associated both with microglia and astrocytes in necrotic areas. Existing data concerning C5b-9 complex is more controversial, with some authors finding no deposition of this factor in ischemic brain tissue (Pedersen et al., 2009), while others reporting increased C5b-9 and C3d immunoreactivity in infarcted areas (Lindsberg et al., 1996). Additionally, immunopositivity for the regulators CD59 and CD55 (Table 1) which is present in healthy controls, is undetectable in ischemic brains (Pedersen et al., 2009), suggesting that down-regulation of these molecules may contribute to ischemic pathology.

Additional information concerning the role of key complement mediators in brain ischemia comes from experimental animal studies. The earliest studies were performed using cobra venom factor (CVF) that induces non-selective but total complement depletion. Rats treated with CVF 1 day before transient middle cerebral artery occlusion (tMCAo), showed higher reactive hyperaemia and better preservation of somatosensory evoked potentials when compared to vehicle-treated rats (Vasthare et al., 1998). A subsequent study demonstrated reduced cerebral infarct volume and atrophy in adult and neonatal rats following CVF administration (Figueroa et al., 2005). In contrast, other studies testing the efficacy of prophylactic CVF administration failed to show any protective effect in a thromboembolic stroke model in rabbits (Lew et al., 1999) or in an hypoxia/ischemia model in immature rats (Lassiter et al., 2001). The species selection and the differences in stroke models may explain, in part, these discordant observations. However, these data also suggest that full complement depletion may not be an optimal therapeutic strategy and that more targeted manipulation aimed at inhibiting/deleting selective complement components should be evaluated. To this end, studies concerning the inhibition of downstream-cascade complement proteins have been conducted to elucidate their specific contribution in mediating brain damage after ischemia. C3−/− ischemic mice showed better neurological scores, reduced ischemic volume, granulocyte infiltration and oxidative stress when compared to WT mice subjected to cerebral ischemia, confirming the deleterious effects of complement system activation in this condition (Atkinson et al., 2006; Mocco et al., 2006a). Furthermore, the expression of C3aR has been shown to increase after cerebral ischemia (Barnum et al., 2002) and its pharmacological inhibition with a C3aR antagonist conferred protection of anatomical damage associated with reduced endothelial ICAM-1 staining (Ducruet et al., 2008).

The precise role of C5 in brain ischemic injury remains unclear. Recent *in vitro* data has shown that brain ischemia, mimicked by oxygen-glucose deprivation, induces the cleavage of the C5 expressed by neurons leading to apoptotic cell death signaling (Pavlovski et al., 2012). *In vivo* studies have shown disparate results depending on post-stroke evaluation time points. Twenty-four hours after ischemic onset, Mocco et al. found no effect on neurological scores or ischemic volume in C5 knock-out mice (C5−/−) when compared to WT mice (Mocco et al., 2006a). Conversely, Arumugam et al. reported improved functional outcome with reduced brain damage in C5−/− compared with their WT littermates, when assessed 72 h after injury (Arumugam et al., 2007). Brain expression of C5aR increases in mice after cerebral ischemia (Barnum et al., 2002) and although the administration of C5aR antagonist elicited only a slight improvement in neurological deficits and infarct volume in 60 min tMCAo mice (Arumugam et al., 2007), more clear cut protective effects on neurological deficits and infarct volume were observed after 45 min tMCAo (Kim et al., 2008).

The deleterious role of terminal complement pathway activation has been assessed in experimental stroke models using a variety of strategies. The first was to investigate the susceptibility of C6 knock-out mice (C6−/−) to ischemic brain damage. No differences concerning either neurological deficits or ischemic damage were observed in C6−/− compared to WT mice (Elvington et al., 2012). The second strategy was aimed at studying the susceptibility to ischemia in CD59 knock-out mice (CD59−/−), which lack this important endogenous membrane-bound inhibitor of C5b-9 formation (see Table 1). Three days after 30 min tMCAo, CD59−/− showed increased infarct volume, greater neurological deficits and increased brain swelling compared to injured WT mice, indicating that CD59 may exert a protective role by inhibiting the C5b-9 assembly on damaged target cells. In contrast, there were no differences on those outcomes between the two strains when the mice were subjected to 60 min tMCAo followed by 2 days of reperfusion, despite the increase in the number of terminal-dUTP-nick end labeled (TUNEL)-positive cells in CD59−/− compared to WT (Harhausen et al., 2010). In addition, the differences observed were restricted to male mice, while no effects were found in female mice (Harhausen et al., 2010). The most recent strategy has been to investigate the role of CD55 regulator (see Table 1) after ischemic brain insults. Primary cortical rat neurons were exposed to hypoxia-like conditions, mimicked by NaCN exposure, and incubated with or without the complement inhibitor CD55. The complement inhibitor prevented dendritic spine loss induced by hypoxia and increased cell viability following the hypoxic insult. In addition, CD55 treatment attenuated the increase of C3 and C3aR neuronal expression, as well as the generation of C3a and C5b-9 triggered by hypoxemia (Mack et al., 2006; Wang et al., 2010). Taken together, these different strategies combine to suggest an involvement of the terminal complement pathway activation in inducing cerebral damage after ischemia.

#### Distinct roles of activation pathways

Further research has been performed on the initiators of the complement cascade to better clarify the specific contribution of the different activation stimuli. It has been demonstrated that C1q, the initiator of the CP, is present on neuronal cell bodies and in cellular debris beginning 6 h after tMCAo (Mack et al., 2006). Furthermore, widespread C1q biosynthesis has been detected in rat microglia, but not in astrocytes or neurons, 24 h after global cerebral ischemia (Schäfer et al., 2000). Nevertheless, we and others have demonstrated that C1q knock-out (C1q−/−) mice were not protected against brain ischemia (De Simoni et al., 2003; Mocco et al., 2006a), suggesting that this component of the complement cascade does not directly induce post-ischemic neuronal damage. Conversely, neonatal C1q−/− mice subjected to hypoxic/ischemic brain injury showed a reduction of infarct volume, neurological deficits, C3 deposition and granulocytes infiltration in infracted areas compared to WT mice (Ten et al., 2005). The apparent discrepancy regarding the C1q role after adult vs. neonatal ischemic injury might be explained by differing age-dependent complement susceptibility. Indeed, it has been demonstrated that a developmentally-related increase in C1q, FB, C3, C4 and C5 expression occurs up to 24 months in mice (Reichwald et al., 2009; Stephan et al., 2013). Although no data are available regarding the expression of C1q, C1q receptors or complement regulators in newborn mice, it is known that C1q is extremely important during neuronal wiring and synapse elimination (vide infra and see Stephan et al., 2012), suggesting a higher susceptibility/sensitivity to C1q-mediated effects in newborn vs. adult neurons.

C1-INH, with its ability to bind and inactivate C1r and C1s, has been suggested to be one of the major regulators of the CP (Ziccardi, 1981). However, subsequent studies (Davis et al., 2010) have demonstrated that this molecule is able to inhibit both the classical and the LPs. Furthermore, it has been shown that C1-INH possesses a greater ability to inactivate MASP-2 than classical proteases, suggesting that the LP might be a more relevant target for C1-INH compared to the CP (Kerr et al., 2008). Additional studies have shown that exogenous administration of C1-INH was protective in a variety of brain ischemia experimental models (Heimann et al., 1999; Akita et al., 2003; De Simoni et al., 2003, 2004; Storini et al., 2005; Gesuete et al., 2009; Heydenreich et al., 2012). We have reported that mice subjected to cerebral ischemia and treated with C1-INH showed reduced ischemic volume, neurological deficits, degenerating cells and leukocytes infiltration in the damaged parenchyma when compared to saline-treated ischemic mice (De Simoni et al., 2004). The protective effects elicited by C1-INH administration were associated with reduced mRNA expression of endothelial adhesion molecules, including P-selectin and ICAM-1, normally induced by ischemic injury (Storini et al., 2005). More recently, Heydenreich et al. reported that C1-INH administration resulted in a significant reduction of BBB leakage compared to saline in ischemic mice (Heydenreich et al., 2012). In order to better understand the molecular targets of C1-INH, we analyzed the effect of C1-INH administration in C1q−/− mice subjected to cerebral ischemia. Although adult C1q−/− mice were not protected from ischemic injury, C1-INH was effective in reducing the ischemic volume in C1q deprived mice, suggesting that C1-INH protective effects are not mediated by the CP. Taken together, this recent evidence supports the hypothesis for a central role of LP in C1-INH mediated protection, since: (1) C1-INH binds MBL with high affinity *in vitro*, (2) C1-INH and MBL co-localize on ischemic endothelium *in vivo*; and (3) C1-INH administration reduces the levels of functional MBL/MASP-2 complexes in the plasma of ischemic mice (Gesuete et al., 2009).

Detailed analysis of the MBL gene in humans reveals that a surprisingly high percentage of individuals (15–30% of the Caucasian population) carry MBL polymorphisms leading to low circulating MBL levels. Notably, in 2 independent studies, MBL deficiency has been associated with favorable outcome in stroke patients (Cervera et al., 2010; Osthoff et al., 2011) providing an additional rationale for the development of further studies to better understand the specific pathogenic role of MBL and of the other LP activators in stroke. In mice subjected to cerebral ischemia, MBL deficiency was associated with reduced neurological deficits, brain lesion and C3 deposition compared to WT mice during the acute post-injury phase (Cervera et al., 2010; Orsini et al., 2012). However, 7 days after the insult, MBL knockout mice (MBL−/−) did not show any evidence of neuronal protection, indicating that MBL may be necessary during tissue repair processes (Ducruet et al., 2011), suggesting that transient MBL inhibition may be a desirable and efficacious therapeutic strategy. Recently, we have demonstrated that pharmacological inhibition of MBL is highly protective in models of cerebral ischemia, possessing a wide window of efficacy both in mice and rats. We have reported that: (1) treatment with Polyman2, a dendrimeric molecule binding MBL with high affinity, induced a reduction of neurological deficits and ischemic volume when administered up to 24 h after induction of injury in mice; and (2) a functional inhibitory neutralizing antibody against MBL induced a long-lasting (up to 1 month) reduction of neurological deficits and ischemic volume when administered 18 h after ischemia in rats (Orsini et al., 2012). In addition, we have demonstrated that MBL was deposited specifically along the luminal side of ischemic vessels, up to 48 h post-injury. We hypothesize that the wide therapeutic window observed may be the result of targeting this long-lasting pathogenic cascade within ischemic endothelium (Figure 2). More recently, it has been proposed that MBL contributes to cerebral damage by promoting local microvascular thrombosis after ischemia in mice (de la Rosa et al., 2014). In these studies, MBL−/− mice showed significantly improved recovery of regional blood flow at 6 h and reduced fibrinogen levels in the ischemic tissue 24 h after tMCAo when compared to ischemic WT mice. Moreover, the administration of argatroban, a thrombin inhibitor, significantly reduced neurological deficits and ischemic volume in ischemic WT but not in MBL−/− mice, suggesting that the deleterious effects of MBL in the ischemic brain are partially mediated by thrombin activation. Additionally, MASP-1 and thrombin both share common targets, including PARs, on endothelial cells (Megyeri et al., 2009), suggestive of close interplay between LP and extrinsic pathway. The existing data underscore the need for further elucidation of the specific interaction between complement and coagulation systems and its relevance to injury progression in acute brain injury.

As previously stated, in addition to MBL, other activators of LP like ficolins and CL-11 exist that may be relevant in stroke. Available evidence indicate that among the currently-identified LP initiators, ficolin-3 exhibits the highest blood concentration and activation capacity in humans (Hummelshoj et al., 2008). In ischemic stroke patients, serum levels of ficolin-2 and ficolin-3 have been shown to be significantly decreased compared to non-ischemic controls, indicating that ficolins may be consumed during the acute phases of ischemic pathology. Perhaps more importantly, only ficolin-3 levels inversely correlate with the stroke severity at admission (7–8 h) and with outcome at 3–4 days (Füst et al., 2011), suggesting that ficolin-3 contributes to the pathogenic processes of cerebral ischemia. We have recently demonstrated that the LP pathway is activated after aneurysmal subarachnoid hemorrhage (SAH) in patients, and that plasma concentrations of ficolin-3 reflect both the severity of brain injury evaluated by clinical and structural parameters and the extent of SAH-associated brain complications (Zanier et al., 2014). In this rare stroke subtype characterized by the bleeding of an intracranial aneurysm, brain ischemia is generally believed to be the main determinant of unfavorable outcome in the acute phase, occurring as result of initial intracranial bleeding and/or at delayed stages as a consequence of vasospasm. One explanation for the decrease of ficolin-3 and functional LP activity is the consumption of this protein in the course of SAH-related events. LP initiators can recognize and bind damage-associated molecular patterns exposed by dying or reversibly damaged cells through their carbohydrate recognition domain. After stroke, injured endothelial cells may change their glycosylation profile, thereby becoming the earliest suitable targets for LP initiators. Overall, these data argue for a major role of the LP, rather than the CP, in complement-mediated toxic effects after stroke and support the concept that the LP may be selectively targeted to control injury progression in brain ischemia.

The contribution of the AP following brain ischemia is, to date, poorly understood. The major difficultly in assessing the contribution of the AP to ischemic damage is that its main activating protein C3, belongs not exclusively to the AP but also to CP and LP. However, recent strategies have been developed to obtain selective AP inhibition. Elvington et al. (2012) recently employed both FB knock-out mice (FB−/−) and the administration of CR2-fH, a specific inhibitor of the AP, in WT mice and reported that FB−/− and CR2-fH-treated mice displayed significant improvement in neurological scores, smaller ischemic volume, reduced P-selectin expression, neutrophil infiltration and microthrombi formation compared to WT or vehicle-treated animals. Interestingly, FB−/− and CR2-fH-treated mice showed less pronounced protective effects when compared to mice knock-out for both C1q and MBL (C1q/MBL−/−) or WT mice treated with CR2-Crry, which inhibits not only the alternative but the whole activation pathways. In addition, FB−/− and CR2-fH-treated mice, but not C1q/MBL−/− mice, showed increased brain C3d deposition, supporting the hypothesis that the AP is not sufficient *per se* to initiate post-ischemic complement activation (Elvington et al., 2012). Thus overall the AP does not initiate full complement activation but appears to contribute to the propagation of cerebral injury via amplification of the other cascades.

### Traumatic brain injury

#### Evidence of complement system activation

The involvement of the complement system in TBI began to be studied in the early 1990s. Although our current knowledge is limited when compared to that of stroke, evidence has accumulated from both clinical and experimental studies allowing for the identification of critical complement-associated factors as mediators of the pathophysiological sequelae of TBI.

A role for complement after TBI in patients has been suggested by studies of cerebrospinal fluid (CSF; Kossmann et al., 1997; Stahel et al., 2001) and cerebral contused tissue (Bellander et al., 2001; Longhi et al., 2014). Following TBI, C3 and FB levels were 20 and 4 times higher in CSF than those found in controls, respectively (Kossmann et al., 1997). Moreover, C5b-9 levels were 1800-fold increased in TBI patients compared to non-injured controls, suggestive of full activation of complement in TBI (Stahel et al., 2001). Additionally, in contused brain tissue, immunoreactivity for complement C3b, C3d and C5b-9 was increased on neuronal cell surfaces (Bellander et al., 2001), while no staining was detected in control tissue. These observations suggest that complement activation occurs in brain cells following TBI, possibly leading to an exacerbation of cerebral damage after the initial (primary) injury. Extensive BBB damage in patients following moderate to severe TBI may suggest that the major source of complement factors may be the vascular compartment, rather than intraparenchymal expression (Kossmann et al., 1997).

Additional evidence for the role of complement in TBI comes from several experimental animal models. The first study on the effect of global complement inhibition in TBI employed soluble CR1 (sCR1), a molecule that inhibits all complement activation pathways by preventing the C3-convertase formation. Rats pre-treated with sCR1 displayed a 41% reduction in neutrophil extravasation following TBI, suggesting that complete complement system inhibition may attenuate vascular permeability (Kaczorowski et al., 1995). Additionally, mice over-expressing soluble Crry (sCryy, see Table 1), a C3 convertase inhibitor, showed reduced BBB leakage and neurobehavioral deficits after weight drop brain injury when compared to brain-injured WT mice (Rancan et al., 2003). In this study, exogenous administration of Crry-IgG (Crry fused with IgG1Fc for bioavailability purpose) in brain-injured WT mice resulted in an attenuation of neurological deficits at 4 and 24 h after TBI and a reduction of neuronal loss in the hippocampus at 4 h compared with vehicle-treatment (Rancan et al., 2003). Treatment with Crry-IgG not only prevented C3 convertase activation, but also induced an up-regulation of both complement-regulatory genes (C1-INH, CD55 and CD59, see Table 1) and the anti-apoptotic Bcl-2 gene (Leinhase et al., 2006). Although Crry may represent a promising pharmacological treatment in the early phase after TBI, further studies are needed to elucidate its protective mechanism of action (Leinhase et al., 2006). Thus, similarly to what found in stroke models, also in TBI C3 convertase may represent a therapeutical target.

The specific role of C3 in TBI has been assessed in C3−/− mice subjected to cortical controlled impact (CCI) brain injury. Although brain-injured C3−/− mice did not differ from brain-injured WT with respect to pre- or post-injury motor and cognitive performance and brain lesion size after trauma, neutrophil recruitment was shown to decrease in these knock-out mice by approximately 50% (You et al., 2007). Additional effects were observed in C3−/− mice subjected to a traumatic cryoinjury model, which mimics several aspects of the TBI pathology, including BBB opening and brain edema. Using this model, investigators reported decreased lesion volume and vascular damage associated with reduced hemorrhage and neutrophil recruitment sustained by a diminished proinflammatory gene expression (RANTES, Eotaxin, MCP-1 and migration inhibitory factor, MIF) in brain-injured C3−/− mice (Sewell et al., 2004). Although these contrasting data do not permit a clear understanding of the exact role of C3 after TBI, the provocative observations, to date, argue for continued work in this area.

The effect of TP over-activation has been studied in experimental TBI using multiple approaches that have consistently demonstrated a detrimental role for this pathway in TBI. In C5−/− mice or in WT mice treated with C5aR antagonist, a significant reduction of neutrophil extravasation after brain cryoinjury was observed when compared to brain-injured control mice. Since the degree of reduction was incomplete (only reaching 35%), other complement chemoattractant molecules (e.g., C3a) may play a more predominant role in regulating this process in TBI (Sewell et al., 2004). Furthermore, after weight-drop TBI, administration of a C5-binding protein complement inhibitor (OmCI, administered up to 15 min after injury), which blocks the generation of both C5a and C5b-9 (Fluiter et al., 2014), reduced neurological deficits, weight loss, C9 immunostaining and microglia/macrophage activation compared with vehicle treatment (Fluiter et al., 2014). OmCI treatment was also associated with decreased neuronal apoptosis and axonal loss. Since protective effects of OmCI treatment are known to be associated with both C5a and C5b-9 inhibition, in order to better understand the specific contribution of down-stream C5b-9, its synthesis was subsequently prevented via the administration of C6 mRNA antisense oligonucleotide. Similar neuroprotective results were obtained to those observed after OmCI treatment (Fluiter et al., 2014). These data highlight the potential role of C5b-9 (vs C5/C5a) as a major mediator of complement-mediated damage after TBI. However, to date, a selective C5 inhibitory strategy has not been evaluated experimentally using a clinically relevant TBI model. The role of C5b-9 formation has also been investigated using CD59−/− mice (see Table 1). Following weight-drop brain injury, CD59 gene expression was observed to be up-regulated in WT mice, suggestive of the activation of a neuronal protective mechanism against lysis. Brain-injured CD59−/− mice showed increased neurobehavioral deficits and neuronal cell death when compared to brain-injured WT mice, suggesting that CD59 plays a direct role in protecting the brain after TBI, possibly by inhibition of C5b-9 formation, as previously described for stroke (Stahel et al., 2009). Overall, these studies underscore a role for full complement activation in the exacerbation of inflammatory processes leading to neurobehavioral and anatomical damage after TBI and brain ischemia.

#### Distinct roles of activation pathways

Beyond our understanding of the relevance of the end-stage complement components in the pathobiological sequelae of TBI, additional studies have begun to address the significance of the activation of specific complement pathways. C4 is an intermediate complement substrate belonging to both CP and LP activation. Knock-out mice for this complement factor (C4−/−) showed improved motor deficits and less post-injury damage after CCI brain injury when compared to injured WT. Similarly, administration of human C4 reversed the motor recovery in C4−/− mice. These data, demonstrating that C4 impairs the recovery of posttraumatic motor deficits and contributes to brain tissue damage (You et al., 2007), suggest that CP and LP are both involved in the pathogenesis of TBI.

In contused tissue from TBI patients, C1q immunoreactivity was found on microglia/macrophage and astrocyte cell surfaces close to the border zone of brain contusions (Bellander et al., 2001). In mice, C1q gene expression increased both at 1 and 4 days after CCI. However, C1q−/− mice did not show an amelioration of neurobehavioral deficits and a reduction of tissue damage after brain trauma compared to injured WT mice suggesting that inhibition of C1q does not lead to neuroprotective effects in TBI (You et al., 2007). These observations support those observed following cerebral ischemia and suggest that C1q does not represent a major target for treatment of acute brain pathology following CNS injury.

Similarly to what has been reported for stroke, C1-INH (Table 1) has been revealed to possess neuroprotective properties in TBI. Mice injected with C1-INH at 10 min or 1 h after CCI brain injury showed a significant and long lasting improvement of motor function over 4 weeks post-injury. Furthermore, mice treated at 10 min showed improved cognitive outcome with a concomitant decrease in lesion volume. These data demonstrate that C1-INH may be protective via the modulation of early mechanisms of secondary damage after TBI (Longhi et al., 2009). Insight concerning the putative mechanisms of action of C1-INH, obtained from experimental stroke studies (vide supra) further supports a focus on the role of LP in TBI. We therefore investigated the MBL presence in both human and experimental TBI. In TBI patients, we demonstrated that MBL immunostaining was present inside and around brain vessels in contused brain tissue removed both early (<6 h) or late (4–5 d) following TBI. Conversely, MBL staining was absent in control patients (Longhi et al., 2014). Similarly, in mice subjected to CCI brain injury, MBL was distributed inside and around brain vessels, visible up to 1 week after CCI, possibly representing a long lasting event after trauma (Longhi et al., 2014). The effect of MBL deletion has been investigated in MBL−/− mice who showed increased degenerating cells in hippocampus 6 h after mild CCI brain injury with an exacerbation of neurobehavioral deficits 1 week post-injury compared to brain-injured WT mice (Yager et al., 2008). Conversely, when studied up to 5 weeks after severe CCI brain injury, MBL−/− mice displayed better neurobehavioral outcome beginning from the second up to the forth week after injury when compared to WT. These behavioral improvements were associated with reduced neuronal cell loss at 5 weeks (Longhi et al., 2014). These findings suggest that MBL absence may lead either to attenuation of mechanisms of secondary brain damage or to the activation of reparative/regenerative processes that occur at more chronic time points after injury (Longhi et al., 2014). Similar to what has been described in models of stroke, increasing evidence highlight the major contribution of the LP, rather than the CP, in mediating secondary damage after TBI and suggest that LP initiators are a viable therapeutic target for future study to limit tissue damage and cell loss following acute CNS injury.

Similar to studies in brain ischemia, few studies have been performed regarding the role of the AP in TBI, to date. FB−/− mice were reported to exhibit a robust decrease of C5a serum levels at 4 h, 24 h and 7 d after weight-drop brain injury, suggesting that AP activation contributes to overall complement activation. In addition, FB−/− mice showed less neuronal death compared to brain-injured WT at 7 days post-injury (Leinhase et al., 2006). Additional studies have investigated the effect of anti-factor B antibody administration which effectively reduces the activation of the AP. Treatment with this compound conferred protection against TBI-induced neuronal cell death, but did not improve neurological deficits when compared to vehicle treatment. This discrepancy was likely due to the lack of sensitivity of the neurological tests employed to detect subtle changes in behavioral performance (Leinhase et al., 2007). In summary, activation of the AP in the setting of TBI appears to have a detrimental effect on pathological and neurobehavioral outcome, although it remains unclear whether this system plays a key role in triggering the full complement activation.

## Neurodegenerative disorders: the evidence of a delicate balance between complement mediated protective and toxic effects

Neurodegenerative disorders are defined as hereditary or sporadic conditions caused by progressive loss of neuronal structures and functions, until neuronal cell death. Many neurodegenerative conditions, including Alzheimer’s disease (AD), Parkinson’s disease (PD) and amyotrophic lateral sclerosis (ALS), occur as a result of this progressive neurodegenerative processes. Despite they arise from different molecular aberrations, they show important similarities in the pathogenic cellular mechanisms (e.g., protein misfolding, defective protein degradation pathways and mitochondrial dysfunction) and in the immune response (complement system and immune cell activation associated with inflammation). Currently there are no drugs able to slow the progression of these disorders.

Among the neurodegenerative disorders, AD is the most common one. Thirty six million people worldwide were estimated to be living with dementia in 2010, a number expected to reach 115 million people by 2050 (Hugo and Ganguli, 2014). We will focus on the complex, versatile role of the complement system in AD outlining emerging evidence that show either beneficial or detrimental effects of this system depending on the disease stage. Additionally we will discuss the limited data available on PD and ALS pathology. A deeper knowledge on the role of the complement system in neurodegenerative disorders may lead to new therapeutic options.

### Alzheimer’s disease

#### Evidence of complement system activation

Since the early 1980s, the full range of classical and AP activation, involving all steps of the cascade through TP, has been described to be associated with AD (Meraz-Ríos et al., 2013).

In humans, multiple isoforms of complement factors have been reported to be present in the CSF of AD patients. Differences in patterns of complement factor expression between normal controls and AD patients have also been reported at different stages of the pathology, suggestive of a distinctive temporal pattern of complement protein expression in relation to amyloid-β (Aβ) plaque deposition and Aβ plaque maturation. CSF levels of C1q, C3d and C4d have been found to increase during the early stages of AD during Aβ formation, showing a positive correlation with total plaque number from very mild to severe clinical cases (Finehout et al., 2005; Wang et al., 2011; Daborg et al., 2012). In post mortem studies comparing young, middle aged and old cases, similar results have been obtained (Stoltzner et al., 2000), documenting increased brain immunoreactivity for complement proteins with age (Stoltzner et al., 2000; Veerhuis et al., 2003; Fonseca et al., 2004a; Zanjani et al., 2005). The CP initiator C1q has been found to be up-regulated up to 80-fold in areas of human AD brains showing pathological neurodegeneration (Yasojima et al., 1999), a relevant observation in view of the fact that C1q is known to bind directly Aβ fibrils (fAβ) and neurofibrillary tangles (Rogers et al., 1992; Jiang et al., 1994; Shen et al., 2001; Webster et al., 2001; Veerhuis et al., 2003), underscoring the need for a deeper understanding of the C1q specific role in AD pathology.

#### Early vs. late disease stages

Manipulations of the complement system in experimental models have permitted the investigation of the role of the complement components in AD. Recent *in vitro* and *in vivo* data have highlighted a neuroprotective role of C1q. A direct positive effect against Aβ fibrillar and oligomeric-induced neuronal death in primary cortical neurons was demonstrated (Pisalyaput and Tenner, 2008; Benoit et al., 2013). *In vivo* studies using AD transgenic mouse models (one expressing the mutant form of human amyloid precursor protein, hAPP and a second expressing mutated Tau and/or presenilin 1) have been used to understand the role of complement system in AD pathology. In these studies, during the early stages of the disease, C1q induced a potent cascade of neuroprotective gene expression via the activation of the transcription factor cAMP response element-binding protein (CREB) and by increasing the expression of downstream pro-survival effectors (low density lipoprotein receptor-related protein 1B—LRP1B and G protein-coupled receptor 6—GPR6) (Benoit et al., 2013). Conversely, at later stages of AD progression, during Aβ accumulation, C1q−/− AD mice showed a significant decreased microglial activation (50–60%) surrounding the Aβ plaques accompanied by elevated expression of neuronal markers with subsequent protective effect on neuronal integrity (Fonseca et al., 2004b). Taken together, these data are suggestive of a protective role for C1q in the early stages of AD, prior to Aβ deposition, converting to a pathogenic role in association with toxic events over time in the more chronic phases of the disease (late stages).

Similar to C1q, increased C3 CSF levels in advanced AD patients (Finehout et al., 2005; Wang et al., 2011; Daborg et al., 2012) and C3 brain expression in aged AD transgenic mice (Wyss-Coray et al., 2002; Zhou et al., 2008; Reichwald et al., 2009) have been reported. The effects of experimental manipulation of C3 on Aβ deposition, neuronal damage and activation of inflammatory cells have been investigated by multiple approaches at more chronic stages of AD pathology. Transgenic AD mice (over-expressing hAPP and TGF-β) were found to have elevated C3 brain levels associated with reduced Aβ accumulation (Wyss-Coray et al., 2002). Inhibition of C3 convertase formation by the transgenic expression of the sCrry gene, leading to loss of opsonizing effect of C3b, was also shown to result in 2 to 3-fold increase of Aβ accumulation and subsequent neurodegeneration (Wyss-Coray et al., 2002). Subsequently, C3−/− transgenic AD mice showed an age-associated increase in cerebral Aβ deposition and neuronal loss compared with C3-sufficient AD mice (Maier et al., 2008). It is known that C3 products may act as chemoattractant factors for microglial cells involved in the clearance of Aβ protein deposits and C3 deficiency/inhibition has been shown to consistently impair monocyte/macrophage phagocytic capability, likely contributing to Aβ protein accumulation (Maier et al., 2008). These studies demonstrate that the absence/inhibition of the central complement component C3, accelerates AD-like Aβ plaque pathology with aging once plaque pathogenesis is underway and suggest that complement C3 may play an important role in maintaining tissue homeostasis (vide infra). The mechanism(s) underlying the biological importance of increased expression of C3 protein observed both in AD humans and mice at delayed stages remains unclear and may represent an attempt to protect the brain via C3 up-regulation rather than a byproduct of neuronal damage (Maier et al., 2008).

In contrast to the data available on C3, data concerning TP products consistently suggest that they exert detrimental effects during the pathological progression of AD (Yao et al., 1990). In AD patients, an increase in the terminal components (C9 and C5b-9) have been reported only in severe AD (Zanjani et al., 2005; Loeffler et al., 2008). During plaque accumulation, the receptors for C5a (C5aR) have been shown to increase and colocalize with neurofibrillary tangles in human AD brains (Fonseca et al., 2013). C5aR has been reported to be expressed in astrocytes and microglia and to be up-regulated during neurodegeneration (Woodruff et al., 2010). *In vitro*, the combination of Aβ-protein and C5a activated monocytes/microglial cells induced an increase in proinflammatory cytokines (O’Barr et al., 2001), supporting the hypothesis that Aβ and C5a together can induce a chronic microglia-mediated focal inflammatory response in a synergistic manner. *In vivo*, an age- and disease-associated up-regulation of C5aR on microglia in the proximity of Aβ plaques has been reported in transgenic AD mice (Ager et al., 2010). Inhibition of C5a using a C5aR antagonist, induced a decrease in Aβ plaque burden and microglial activation with a concomitant increase in cognitive performance in transgenic AD mice (Fonseca et al., 2009). This effect does not appear to be mediated by the modulation of C1q and/or C3, which remain unaffected after treatment, but by the selective inhibition of deleterious C5a–C5aR signaling (Ager et al., 2010). Thus, the inhibition of the complement TP during AD progression leads to a substantial improvement in behavioral and histopathological outcome in AD mice, suggesting that pharmacological manipulation of this pathway may be a novel strategy to treat neurodegeneration.

Little is known concerning the involvement of LP in AD. Studies showing lower MBL levels in CSF of AD patients when compared to controls (Lanzrein et al., 1998), together with more recent evidence that MBL deficiency in humans is associated with AD risk (Sjölander et al., 2013), underscore the need for further studies to better characterize the role of this pathway in AD pathogenesis and progression.

Thus, AD represents an excellent paradigm to explore the differential role of specific complement factors in relation to time and degree of neuropathological injury. The available data indicate that the complement system plays a dual role in AD pathogenesis and progression. Beneficial effects of complement activation occur during the early stages of AD (Fonseca et al., 2004b; Pisalyaput and Tenner, 2008; Benoit et al., 2013), possibly contributing to Aβ plaques clearance by microglia through complement-dependent opsonization (mediated by C1q, C3b) (Alexander et al., 2008). Conversely, during the more chronic phases of AD progression, complement activation appears to transition to a deleterious role, contributing to neurotoxicity with subsequent exacerbation of the inflammatory reaction at the site of injury (Alexander et al., 2008).

### Parkinson’s disease and amyotrophic lateral sclerosis

Evidence also exists for complement involvement in PD and ALS. In PD patients, increased complement activation (through formation of C5b-9), has been identified in Lewy bodies (LBs, intraneuronal fibrillar aggregates containing a high concentration of α-synuclein) and in oligodendroglia in the substantia nigra, as well as in serum and CSF of patients with either sporadic or familial PD (McGeer and McGeer, 2004; Goldknopf et al., 2006; Wang et al., 2011; Depboylu et al., 2011a; More et al., 2013). Similar to what has been observed in PD, increased complement components in serum (C3c, C3d, FH), in CSF (C4d) (Tsuboi and Yamada, 1994; Goldknopf et al., 2006), as well as in affected postmortem spinal cord tissue (C1q/C2/C4/C3/MAC) have been reported in ALS patients (Grewal et al., 1999; Sta et al., 2011). In experimental rodent models of ALS pathology (one expressing ALS-causing mutations in superoxide dismutase SOD1 and a second engineered to be deficient in the low molecular weight neurofilament- NFL subunit protein) similar findings have also been reported in spinal cord (Lobsiger et al., 2007; Woodruff et al., 2008; Humayun et al., 2009; Takeuchi et al., 2010; Heurich et al., 2011; Lee et al., 2013) and along peripheral nerves (Chiu et al., 2009). Increased expression of C1q reported in brain tissue from humans and mouse models of both PD (Depboylu et al., 2011a,b) and ALS (Lobsiger et al., 2007; Heurich et al., 2011; Sta et al., 2011) highlights the potential involvement of C1q in neurodegenerative processes via microglial-mediated synaptic elimination. The lack of effect of C1q gene deletion on onset and disease progression in either PD mice (Depboylu et al., 2011b), or SOD1 mutant mice complicates our understanding of the role of C1q in these conditions (Lobsiger et al., 2013) and the exact role of CP in PD and ALS pathogenesis and progression remains poorly understood.

No additional data are available concerning the possible role of other complement proteins in PD. In ALS, however, the role of C3 (Lobsiger et al., 2013) and C4 (Chiu et al., 2009) using gene deletion techniques has been studied in SOD1 mutant mice showing no major effect on outcome. Alternatively, recent evidence suggests that the C5 downstream protein may be a key mediator of complement-mediated neurotoxicity in ALS models. Studies in transgenic SOD1 mutant rats and NFL knock-out mice have reproducibly shown an up-regulation of the major proinflammatory C5aR during disease progression (Woodruff et al., 2008; Humayun et al., 2009). Furthermore, chronic administration of C5aR antagonist in transgenic SOD1 mutant rats was reported to exert beneficial effects on neuronal survival during disease progression (Woodruff et al., 2008). These results indicate that, under stress, local complement signaling might therefore promote damage and motor neuron death in ALS.

## The complement system in homeostasis, plasticity and regeneration

Growing evidence highlights that the versatile functions of the complement system extend far beyond those of immune surveillance and the elimination/neutralization of pathogens and altered host cells. Indeed, recent evidence point to an active involvement of complement in lipid metabolism, angiogenesis, tissue remodeling and maintenance in the CNS (Veerhuis et al., 2003; Ricklin et al., 2010; Rutkowski et al., 2010; Stephan et al., 2012).

### Synaptic remodeling

Plasticity and remodeling of new synaptic circuits characterize the early stages of brain development (Stephan et al., 2012). Molecular and cellular mechanisms underlying synapse refinement during development have been studied using the retinogeniculate system. Early in development, axons from retinal ganglion cells (RGCs) form transient functional synaptic connections with neurons of dorsal lateral geniculate nucleus (dLGN). Subsequently, during the first 2 weeks of post-natal development, the retinogeniculate system circuit is subjected to precise sculpting through the pruning of any overlapping or redundant transient connections and the strengthening of the remaining ones (Hooks and Chen, 2006). The failure of this mechanism results in uncorrected eye-segregation and visual deficits. During this period of synaptic refinement, a close interaction/interplay amongst complement proteins, immature astrocytes and neurons has been revealed to occur. In the postnatal retinogeniculate system, TGF-β released by immature astrocytes, induces in RGCs the up-regulation of C1q which is transported from cell bodies along axons to the dLGN, where it is released to bind transient synapses. C1q is therefore believed to contribute to synapse elimination through direct connection tagging (Eggleton et al., 2000) or through C3b formation (Bialas and Stevens, 2013) that, in turn, opsonizes the target synapses. The contribution of C1q and C3b to synaptogenesis and synaptic remodeling is also supported by their punctate colocalization with pre and postsynaptic markers during normal retinogeniculate development (Stevens et al., 2007). However, the mechanism that drives the opsonization process of synapses destined to be pruned is not fully understood. Two different mechanisms, both claiming a central role of complement opsonin (C1q and C3b) and microglial interplay (Schafer et al., 2012), have been suggested: (1) C1q and C3b, via an unknown selection strategy, only tag the overlapping and weaker synapses, thereby inducing their elimination by phagocytic microglia (Stevens et al., 2007), or (2) C1q and C3b tag all synapses but the more active or stronger ones are, in turn, able to express specific membrane-bound complement inhibitors which confer selective protection against microglial phagocytosis (Kim and Song, 2006; Kim et al., 2008; Stephan et al., 2012). In addition, resident microglia are not only involved in active phagocytosis of tagged synapses but may participate to synaptic modeling through the secretion of C1q. To this end, increased concentrations of C1q have been shown to be present in microglia during the postnatal period that coincides with the peak of synaptic refinement (Fiske and Brunjes, 2000). The involvement of C1q and C3 fragments in brain sculpting is likewise supported by studies in knock-out animals who display an incorrect eye-specific segregation, due to overlapping RGC projections and overabundant excitatory connectivity in the cortex, resulting from defective synapse elimination (Stevens et al., 2007; Chu et al., 2010; Stephan et al., 2012). However, the phenotype of C1q−/− and C3−/− mice also shows residual synapse refinement, suggesting that other molecules may participate in this process, including both major histocompatibility complex I (MHC-I) and neuronal pentraxins (Corriveau et al., 1998; Huh et al., 2000; Bjartmar et al., 2006; Datwani et al., 2009). These findings show that complement proteins are important in cooperating with other pathways to regulate normal synaptic circuit development.

While complement activation and its interaction with microglial cells appear necessary for brain wiring during the postnatal period, recent evidence indicate that when this developmental program becomes aberrant in the immature brain or is recapitulated during adulthood molecular cascades related to neurodegenerative processes may be initiated (Stephan et al., 2012). For example, during the postnatal period, the inappropriate activation of the complement cascade causes profound synapse elimination that leads to neuropsychiatric diseases, such as autism or schizophrenia (Patterson, 2011). Defects in pruning have also been reported to be associated with the development of epilepsy (Chu et al., 2010) or glaucoma (Howell et al., 2011) where C1q and C3 are pathologically up-regulated and cause destabilization of neuronal circuits (see Stephan et al., 2012).

### Complement-mediated effects on cellular waste removal

One of the main “housekeeping” functions of the complement cascade involves the removal of apoptotic cells and the scavenging of cellular debris and immune complexes. Senescent or defective cells typically undergo apoptosis via a non-inflammatory cell death. Normally, apoptotic cell bodies are rapidly removed to avoid the unwanted over-activation of immunological processes, since failure to clear or scavenge dead cells may induce an exaggerated inflammatory reaction against other tissues (Savill et al., 2002; Cole et al., 2006). Apoptosis induces membrane phospholipid and ionic charge changes leading to deposition of innate system effectors (Flierman and Daha, 2007). As for the brain, i*n vitro* studies have shown that C1q binds to neuronal apoptotic cells and activates the CP with subsequent production of opsonizing fragments (C3b and C4b), responsible for apoptotic cell phagocytosis (Cole et al., 2006). Furthermore, biochemical studies have demonstrated that C1q is able to bind IgM, serum amyloid P component (SAP), CRP and pentraxin-3 (PTX3), suggesting their involvement in C1q- mediated apoptotic cell removal (Kishore et al., 2004). The relevance of C1q in the physiological clearance of apoptotic cells has been studied in C1q−/− mice who displayed an impaired elimination of immune complexes and susceptibility to autoimmune disease (Mitchell et al., 2002).

The overall role of phagocytosis is complex and depends on the context. When occurring on the target cell dying by some means such as apoptosis it is believed to be a beneficial activity, preventing the release of damaging and/or proinflammatory intracellular components (referred to as “secondary phagocytosis”, Neher et al., 2011, 2012). However, under certain conditions, such as acute brain injury, complement overactivation may induce microglial cells to phagocyte also viable neurons, thus executing their death (referred to as “primary phagocytosis”) (Neher et al., 2011, 2012). After injury such phagocytic activity may result from exposure of eat-me signals (C3b, C4b and C5b) on otherwise viable neurons as a result of subtoxic and reversible insults, thus contributing to injury amplification.

### Complement-mediated effects on mature brain cell survival and on neurogenesis

*In vitro* studies have shown pro-survival effects of C1q in mature brain cells, under normal conditions. Specifically, rat neuronal cell cultures treated with C1q showed prolonged cell survival associated with higher number of neuronal processes when compared to untreated cells (Pisalyaput and Tenner, 2008). These pro-survival effects of C1q may be due to an up-regulation of cholesterol metabolism and cytoskeleton-related gene expression and to an increase of nerve growth factor (NGF) and neurotrophin-3 protein levels (Benoit and Tenner, 2011). Indirect evidence for a pro-survival action of C3a and C5a have also been presented and C3a and C5a treatment of astrocytes and microglial cell cultures has been shown to up-regulate NGF, suggesting a common pro-survival pathway in the CNS under normal conditions (Heese et al., 1998). Under neurodegenerative conditions, a protective role of C1q and C3 in preserving tissue homeostasis during AD progression has been reported (vide supra).

Complement proteins have been shown to be involved in migration and maturation of stem cells in the CNS under physiological conditions. Neural stem and progenitor cells (NPCs) have been reported to express C3aR and C5aR (Rahpeymai et al., 2006). *In vitro* studies have demonstrated that treatment of NPCs with C3a facilitates their migration and maturation without affecting proliferation (Shinjyo et al., 2009). In addition, it has been reported that mice treated with a C3aR antagonist display reduced neurogenesis in different brain areas, including the subventricular zone (SVZ), hippocampal dentate gyrus and olfactory bulb. Overall, these findings indicate that cellular signaling via C3a positively regulates basal neurogenesis (Rahpeymai et al., 2006).

Complement seems to be involved also in brain injury-induced neurogenesis. After transient ischemia, C3−/− mice showed a significant reduction (by 24%) in neurogenesis (doublecortin positive cells) in the SVZ compared to ischemic WT mice at 7 days after insult. Moreover, the reduction in neurogenesis was independent of either microglia activation or reactive gliosis that remained unaffected in C3−/− compared to WT mice (Rahpeymai et al., 2006). Further evidence suggesting a contribution of C3a to CNS neurogenesis comes from the literature concerning experimental neonatal brain ischemia. Ischemic neonatal mice treated with C3a exhibited improved memory function at 41 days and this effect was abolished in C3aR−/− neonatal mice (Järlestedt et al., 2013). As discussed previously, a large body of evidence has been generated demonstrating the detrimental effects of several complement-activated products after acute brain injury. While these findings suggest a contribution of C3a to ischemia-induced neurogenesis, it should be emphasized that C3 is not a unique/major neurogenic factor since consistent and residual neurogenesis remains after C3 removal. Continued investigation addressing the temporal role of the complement system in mediating neurogenesis in the injured brain is warranted to elucidate how the delicate balance between complement-dependent neurotoxicity and its potential neurogenic effects can be modulated to promote neuroprotection.

## Concluding remarks

Far beyond the view of the complement as a supplementary molecule needed for bacterial lysis, the available data show that the complement cascade is involved in several aspects of brain development, homeostasis, injury and regeneration. The versatility of this cascade in participating in diverse processes in the nervous system under both physiological and pathological conditions appears to be dependent on a fine balance within an intricate network of effectors, receptors and regulators. When the critical factors involved with this system are finely regulated, they participate in the maintenance of brain homeostasis. Conversely, deregulation between activators and regulators leads to aberrant complement activation with subsequent exacerbation of inflammation and worsening of the damage induced by brain injury and neurodegenerative diseases. Importantly, evidence that targeting selective steps of this cascade leads to amelioration of brain injury strongly support the concept of the complement system as an important therapeutic target in brain injury and disease.

## Conflict of interest statement

The authors declare that the research was conducted in the absence of any commercial or financial relationships that could be construed as a potential conflict of interest.

## Abbreviations

AD: Alzheimer’s disease
ALS: amyotrophic lateral sclerosis
AP: alternative pathway
A*β*: Amyloid-*β*
BBB: blood brain barrier
C1-INH: C1 inhibitor
C3aR: C3a receptor
C5aR: C5a receptor (also known as CD88)
CCI: cortical controlled impact
CL-11: collectin-11
CNS: central nervous system
CP: classical pathway
CRP: C-reactive protein
CSF: cerebrospinal fluid
CVF: cobra venom factor
DAMPs: damage-associated molecular patterns
dLGN: dorsal lateral geniculate nucleus
hAPP: human amyloid precursor protein
HUVECs: human umbilical vein endothelial cells
ICAM-1: intercellular adhesion molecule-1
LP: lectin pathway
MAC: membrane attack complex
MASPs: MBL-associated serine proteases
MBL: mannose binding lectin
MCP-1: monocyte chemotactic protein-1
NFL: neurofilament subunit protein
NGF: nerve growth factor
NPCs: neural stem and progenitor cells
PAMPs: pathogen-associated molecular patterns
PARs: protease activated receptors
PD: Parkinson’s disease
RANTES: regulated on activation normal T cell expressed and secreted
RGCs: retinal ganglion cells
SAH: aneurysmal subarachnoid haemorrhage
SOD1: superoxide dismutase 1
SVZ: subventricular zone
TBI: traumatic brain injury
tMCAo: transient middle cerebral artery occlusion
TP: terminal pathway
VCAM-1: vascular cell adhesion protein-1
WT: wild type.
