# Corrigendum: Versatility of the complement system in neuroinflammation, neurodegeneration, and brain homeostasis

**DOI:** 10.3389/fncel.2015.00263

**Published:** 2015-07-07

**Authors:** Franca Orsini, Daiana De Blasio, Rosalia Zangari, Elisa R. Zanier, Maria-Grazia De Simoni

**Affiliations:** ^1^Department of Neuroscience, IRCCS – Istituto di Ricerche Farmacologiche Mario NegriMilan, Italy; ^2^Department of Experimental and Clinical Sciences, University of ChietiPescara, Italy; ^3^Department of Anesthesia and Critical Care Medicine, Fondazione IRCCS Ca' Granda Ospedale Maggiore Policlinico, University of MilanMilan, Italy

**Keywords:** complement system, therapeutic targets, endothelium, stroke, traumatic brain injury, Alzheimer's disease, brain homeostasis

In the funding statement one source of funding was missing. This is now rectified as follows:

## Conflict of interest statement

The authors declare that the research was conducted in the absence of any commercial or financial relationships that could be construed as a potential conflict of interest.

